# Compound heterozygous mutations in *CFTR* causing CBAVD in Chinese pedigrees

**DOI:** 10.1002/mgg3.486

**Published:** 2018-11-18

**Authors:** Bin Yang, Xi Wang, Wei Zhang, Hongjun Li, Binbin Wang

**Affiliations:** ^1^ Department of Urology Union Medical College Hospital, Chinese Academy of Medical Science Peking Beijing China; ^2^ Center for Genetics National Research Institute for Family Planning Haidian, Beijing China

**Keywords:** *CFTR*, congenital bilateral absence of the vas deferens, whole exome sequencing

## Abstract

**Background:**

Congenital bilateral absence of the vas deferens (CBAVD) is an important cause of obstructive azoospermia and male infertility. Mutations of *CFTR* caused the majority of CBAVD cases, and *ADGRG2* was recently identified as a new pathogenic gene. Yet, most of the genetic evidence came from sporadic cases, and only one mutation in *CFTR* can be found in patients.

**Methods:**

In present study, we collected two CBAVD pedigrees, each having two affected male siblings. We performed whole exome sequencing on all patients and validated all potential variants by Sanger sequencing.

**Results:**

We excluded *ADGRG2* variants but identified compound heterozygous variants of *CFTR* in both families (NM_000492.3:c.1210‐33_1210‐6GT[13]T[5] and c.4056G>C;p.Gln1352Cys in pedigree 1, c.592G>C;p.Ala198Pro and c.3717G>A;p.Arg1239= in pedigree 2), which were subsequently validated by direct sequencing. c.1210‐33_1210‐6GT[13]T[5] (also known as IVS8‐T5‐TG13) was a known disease‐causing variant causing the skipping of exon 9 of CFTR and inherited from the proband's mother. p.Gln1352Cys and Ala198Pro were rare or novel in public databases and predicted to be deleterious. The p.Arg1239= was a synonymous variant but located at the end of an exon, which was predicted to alter the splicing pattern.

**Conclusion:**

Our study, in which compound heterozygous variants were identified in two pedigrees, provides more familial evidence that only recessive variants (homozygous or compound heterozygous) in *CFTR* cause CBAVD. Furthermore, whole exome sequencing may be utilized as a useful tool for mutation screening of genes causing CBAVD.

## 1 INTRODUCTION

Cystic fibrosis (CF) is one of the most common autosomal recessive disorders in the Caucasian population but is uncommon in Asians. The relationship between the cystic fibrosis transmembrane conductance regulator gene (*CFTR*, OMIM: 602421) and CF is well‐established (Zielenski & Tsui, 1995), with over 2000 *CFTR* variants identified, as curated by the Cystic Fibrosis Variations Database (https://www.genet.sickkids.on.ca/cftr/app).

Most males with CF are infertile because of obstructive azoospermia (Chillon et al., 1995). Congenital bilateral absence of the vas deferens (CBAVD, OMIM: 277180) is one type of male reproductive tract abnormality that causes 1%–2% of male infertility and up to 6% of obstructive azoospermia (Du et al., 2014). Because almost all infertile CF males exhibit CBAVD, it is widely considered an atypical form of CF and a CFTR‐related disorder (Kaplan et al., 1968).

*CFTR* is located on chromosome 7q31.2 and contains 27 exons. It encodes a glycosylated transmembrane chloride channel that is widely expressed in the epithelial cells of efferent ducts and is responsible for fluid absorption in organs such as the sweat glands, lungs, and vas deferens (Kujala et al., 2007; O'Sullivan & Freedman, 2009). Hundreds of *CFTR* variants have been reported in CBAVD patients, with the four most common being c.1210‐12T[5], p.Phe508del, p.Met470Val, and p.Arg117His (Grangeia et al., 2004); 70%–80% CBAVD patients carry at least one *CFTR* variant (Yu, Chen, Ni, & Li, 2012). There is a population difference of *CFTR* mutation spectrum. The most common variants seen in the Caucasian CBAVD patients were 5T allele, p.Arg117His, and p.Phe508del (Grangeia et al., 2004). However, the p.Phe508del mutation was totally not found in Chinese CBAVD patients (Li et al., 2012; Ni et al., 2012).

c.1210‐12T[5] is the shortest form of the IVS8‐Tn variant with an incomplete penetrance that results in abnormal splicing of intron 8 and skipping of exon 9 in many* CFTR* transcripts (Chillon et al., 1995; Chu, Trapnell, Curristin, Cutting, & Crystal, 1993; Teng et al., 1997); two other forms, 7T and 9T, predominantly generate normal transcripts. Previous studies demonstrated that the penetrance of 5T may depend on the copy number of adjacent TG repeats (Chiang, Lu, Liu, Wu, & Wu, 2009). Different alleles of the (TG)m(T)n locus are associated with variable splicing efficiencies of intron 8 (Cuppens et al., 1998), while the common variant p.Phe508del may impair protein folding and trigger proteasome activity for degradation (Lukacs & Verkman, 2012). The p.Met470Val variant occurs in certain populations and produces the 470Val protein which has a reduced function compared with the 470Met protein (Cuppens et al., 1998). However, the pathogenic role of p.Met470Val remains unclear.

Besides *CFTR*,* ADGRG2* (OMIM: 300572) on chromosome X was recently identified as another disease‐causing gene of CBAVD (Patat et al., 2016). Patat et al. identified three hemizygous *ADGRG2* variants causing truncated proteins in four patients by whole exome sequencing after excluding *CFTR* variants. We replicated these findings in a cohort of Chinese CBAVD patients and also detected another two *ADGRG2* deleterious missense variants in two *CFTR*‐negative patients (Yang et al., 2017), supporting the fact that *ADGRG2* variations may explain a proportion of CBAVD cases.

Previous genetic studies were mostly performed in sporadic cases. Here, we aim to identify the genetic cause of two Chinese pedigrees and report the family‐based genetic findings. Through exome sequencing, we excluded *ADGRG2* variants as the cause and found compound heterozygous variants (NM_000492.3:c.1210‐33_1210‐6GT[13]T[5], c.4056G>C;p.Gln1352Cys and c.592G>C;p.Ala198Pro, c.3717G>A;p.Arg1239= in each pedigree, respectively) in *CFTR* which were subsequently validated by Sanger sequencing.

## 2 MATERIALS AND METHODS

### 2.1 Ethical compliance

This research was approved by the Research Ethics Committee of Peking Union Medical College Hospital, and experiments were performed in accordance with approved guidelines.

### 2.2 Participants

**Figure 1:**
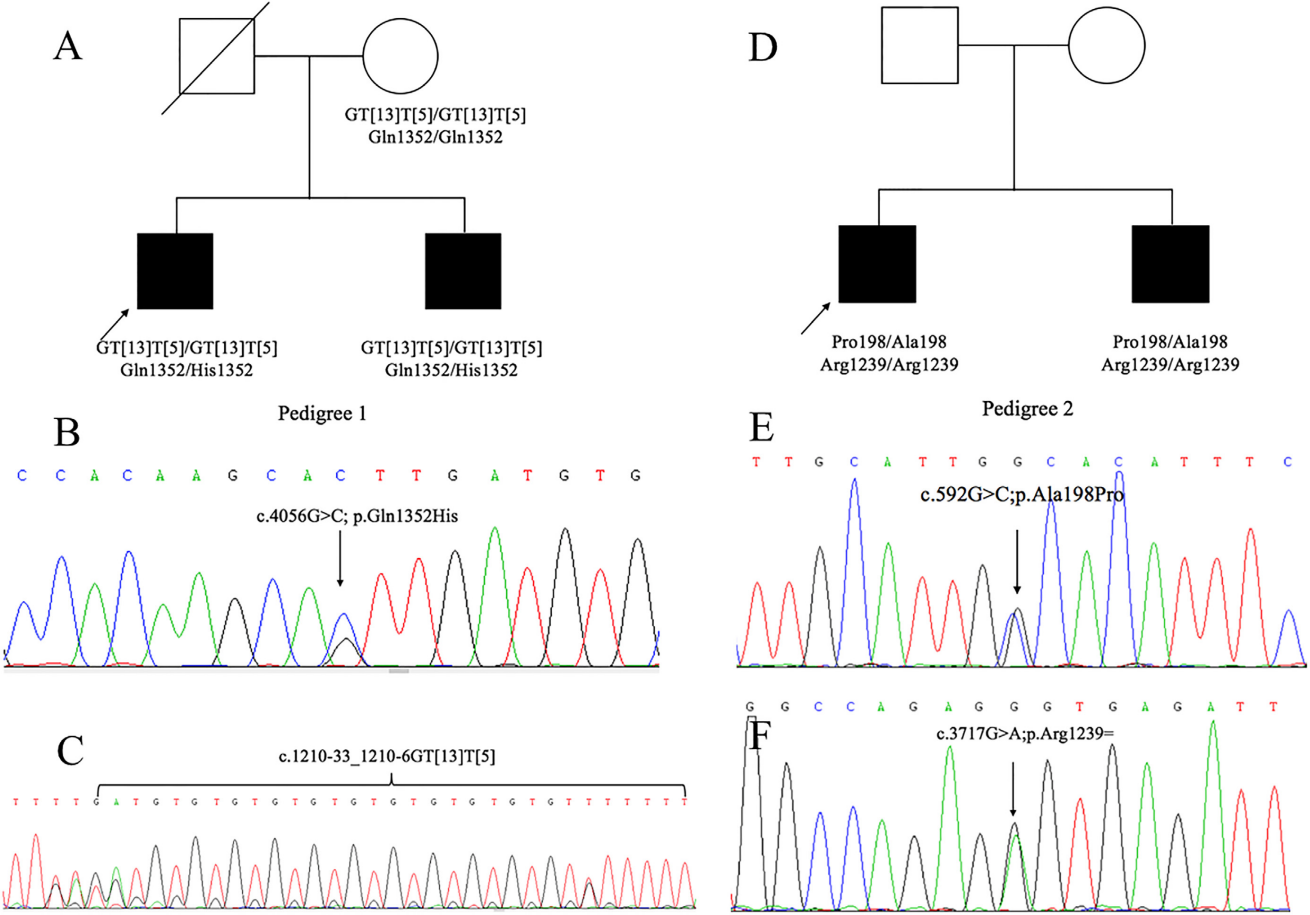
Pedigree structure of the family and Sanger sequencing validation. (a) Family pedigree 1; (b) the c.4056G>C;p.Gln1352Cys variant was inherited from their father; (c) the c.1210‐33_1210‐6GT[13]T[5] variant was inherited from their mother; (d) Family pedigree 2; (e) Sequencing validation of c.592G>C;p.Ala198Pro; (f) Sequencing validation of c.3717G>A;p.Arg1239=

Two Chinese pedigrees with four males diagnosed with CBAVD was recruited from the Urological Department, Peking Union Medical College Hospital (Chinese Academy of Medical Sciences, Beijing, China) (Figure 1a). The parents of both pedigrees did not report any consanguinity. Two male offspring of pedigree 1 were 28 and 24 years old, respectively, and claimed infertility although having regular intercourse after marriage for several years. The two patients of pedigree 2 were 25 and 27 years old, and have been infertile for 3 and 4 years after marriage, respectively. All patients had impalpable scrotal vas deferens, low semen volume (<2.0 ml) and pH value (≤7.0), and decreased seminal fructose but normal serum follicle‐stimulating hormone and luteinizing hormone. They had no classical symptoms of CF except for CBAVD. Ultrasonography showed that their kidneys were normal.

Genomic DNA was extracted from peripheral blood samples (QIAGEN, USA). Written informed consent was obtained from all participants.

### 2.3 Whole exome sequencing and validation

Whole exome sequencing was performed on all patients with all exons captured by the SureSelect Human All Exon V5 Enrichment kit. These then underwent high‐throughput sequencing on the Illumina HiSeq 4000 platform (Illumina, USA), and reads were aligned to the human reference genome (UCSC, hg19) using Burrows–Wheeler Aligner v0.5.9. Single nucleotide variants and insertion/deletions were detected according to GATK best practice, and all variants were annotated by ANNOVAR.

Public variant databases, including the 1000 Genomes Project (1000G), NHLBI ESP6500, and ExAC were used to filter variants with a minor allele frequency >0.01. Three in silico programs, including PolyPhen2, SIFT, and MutationTaster were used to predict the impact of variants on protein function and structure. HSF3 and ManEntScan were used for the online analysis of splicing site.

All variants were validated in the pedigree by Sanger sequencing using the following primers: forward 5′‐GGGAGGTAGCACCAAGGA‐3′ and reverse 5′ ‐GCGTTAGCCACAATCAGC‐3′ for c.4056G>C, forward 5′‐CATAAAACAAGCAT CTATTG‐3′ and reverse 5′‐AGAGACATGGACACCAAATT‐3′ for c.1210‐33_1210‐6GT[13]T[5], forward 5′‐ CATTGCTATGTGCTCCAT‐3′ and reverse 5′‐TTCATCATCATTCTCCC T‐3′ for c.592G>C, forward 5′‐TCTGTGAGCCGAGTCTTT‐3′ and reverse 5′‐TTGTT TGGCAGAATGGAA‐3′ for c.3717G>A. The reaction components and PCR conditions have been reported previously (Du et al., 2014). We used the GenBank reference sequence AH006034.2.

## 3 RESULTS

Whole exome sequencing showed that the patients in each pedigree did not share nonsynonymous variants (missense, nonsense, frameshift, and splicing variants) in *ADGRG2*. Therefore, *ADGRG2* was excluded as causative of infertility. Analysis of the exons and splicing region sequences showed that both patients in pedigree 1 shared a rare missense variant NM_000492.3:c.4056G>C;p.Gln1352Cys. Additionally, in the intron 8 and exon 9 junction region of *CFTR*, they both had two G insertions, which were confirmed by subsequent direct sequencing to be the c.1210‐33_1210‐6GT[13]T[5] variant (also known as IVS8‐T5‐TG13), a known pathogenic variant of CBAVD. In pedigree 2, both patients shared two rare heterozygous variants, one is c.592G>C;p.Ala198Pro and another is c.3717G>A;p.Arg1239=. All variants were validated by Sanger sequencing (Figure 1b,c,e,f).

Co‐segregation analysis of pedigree 1 by Sanger sequencing demonstrated that variant c.1210‐33_1210‐6GT[13]T[5] was inherited from their mother (Figure 1c). Although the father's DNA was unavailable, it was reasonable that the other variant derived from him. Therefore, both heterozygous variants were in trans and in a compound heterozygous state (Figure 1b). However, co‐segregation analysis was not performed in pedigree 2 due to the unavailability of the parents’ DNA. Yet, we assumed they would be in compound heterozygous state, because they are extremely rare or novel in public databases and more likely to be maternally or parentally inherited, respectively.

The p.Gln1352Cys variant is rare in public databases (0.001 in ExAC, 0.004 in 1000G, and absent from ESP) while p.Ala198Pro is totally absent. They are both predicted to be deleterious by all in silico programs and are highly conserved among species. Interestingly, another variant, c.3717G>A;p.Arg1239=, does not change the amino acid but locates at the end of the 22nd exon, adjacent to the splicing donor site. The location of the variant suggests that it may alter the mRNA splicing pattern. Therefore, we used HSF3 and MaxEnt to predict the alteration. The HSF score reduced from 82.52 to 71.94 after mutation, with a reduction of 12.82% over the threshold of 10%. Meanwhile, the MaxEntScan score reduced from 4.44 to −1.25, which greatly reduced by 128.15% beyond the threshold of 30%. Taken together, this variant may abolish the current splicing donor site and use a cryptic site, leading to abnormal mRNA splicing of CFTR.

## 4 DISCUSSION

Cystic fibrosis is one of the most common lethal diseases in the Caucasian population, with an incidence as high as 1 in 2,500 live births (Kerem, Chiba‐Falek, & Kerem, 1997). It is believed to be rare in Asian populations including Chinese, but CBAVD is not. *CFTR* mutation screening, in the coding, promoter or splicing regions, has been performed in Chinese CBAVD individuals in several studies (Bai, Du, Liu, Tong, & Wu, 2018; Du et al., 2014; Li et al., 2012; Lu et al., 2013; Ni et al., 2012), which established a genetic link between *CFTR* and CBAVD in the Chinese population. However, no CBAVD pedigree with compound heterozygous variants of *CFTR* has been reported in the literature to date.

In this study, we described two Chinese pedigrees in which four male offspring were diagnosed with CBAVD without other clinical symptoms of CF. Through whole exome sequencing, we identified c.1210‐33_1210‐6GT[13]T[5] and c.4056G>C;p.Gln1352Cys in both patients of pedigree 1, and c.592G>C;p.Ala198Pro and c.3717G>A;p.Arg1239= in pedigree 2, which were subsequently confirmed by direct sequencing. The compound heterozygous variants in pedigree 1 were previously reported in three unrelated Japanese CBAVD patients (Anzai et al., 2003), supporting their pathogenic role in CBAVD.

The poly‐T polymorphism and TG repeat at the intron 8–exon 9 junction of *CFTR* are commonly studied in individuals with CBAVD. The polymorphism has three different forms, 5T, 7T, and 9T. Population studies found a significantly higher frequency of 5T allele in CBAVD patients compared with general population or healthy men in multiple populations (Asadi, Mirfakhraie, Mirzajani, & Khedri, 2018; Chillon et al., 1995; Gaikwad et al., 2018; Ni et al., 2012). Individuals with the 5T allele may express high levels of aberrant transcript with the skipping of exon 9, which may account for 92% of the total mRNA in the presence of the bi‐allelic 5T variant (Chu et al., 1993). The protein product lacking exon 9 will not contribute to chloride channel activity. The 5T allele is considered to have a variable penetrance because of the observed different phenotypes in its carriers (Danziger, Black, Keiles, Kammesheidt, & Turek, 2004).

Previous studies indicated that the 5T allele has an increased penetrance when combined with higher numbers of adjacent TG repeats (Cuppens et al., 1998). Three different TG repeats are found in cis with the 5T allele (TG11, TG12, and TG13). Functional experiments demonstrated that TG11 and TG12 alleles resulted in a 2.8‐fold and sixfold increase, respectively, in the proportion of *CFTR* transcripts lacking exon 9 compared with the TG10 allele on a T7 background (Cuppens et al., 1998). Longer repeats with shorter poly‐T tracts may lead to incorrect splicing of intron 8 and increase the possibility of exon 9 skipping (Claustres, 2005). 5T‐13TG haplotype, the longest TG repeats in combination with the shortest T repeats, was found in 9.2% and 5.9% of CBAVD patients, but not seen in health men in Chinese and Indian populations, respectively (Gaikwad et al., 2018; Ni et al., 2012), suggesting a pathogenic role of this rare haplotype.

The CFTR protein is comprised of two repeated regions, each containing a transmembrane domain and a nucleotide binding domain. Both regions are linked by a cytoplasmic hydrophilic regulatory domain (Cheng et al., 1991; Zielenski et al., 1991). Exon 9 encodes part of the first nucleotide binding domain, and when absent produces a misfolded and dysfunctional CFTR chloride channel (Delaney et al., 1993). Consequently, functional CFTR protein expression is dramatically reduced in IVS8‐T5‐TG13 variant carriers.

Another variant in pedigree 1, p.Gln1352Cys, was shown to be highly conserved in various species and predicted to be deleterious by online prediction programs. It is located in the second nucleotide binding domain of CFTR, and the substitution of glutamine to histidine is thought to decrease the chloride channel activation efficiency.

In pedigree 2, p.Ala198Pro is not located in any domains of CFTR and seems to be a missense variant with moderate pathogenicity. Another variant, c.3717G>A;p.Arg1239=, is reported only once in ExAC and is a known disease mutation as recorded in HGMD (https://www.hgmd.cf.ac.uk). It was once found with p.Phe508del mutation in a cystic fibrosis patient who had moderate to severe pulmonary disease and pancreatic insufficiency (Cutting et al., 1992). The online prediction indicated that it may alter the splicing pattern after the 22nd exon which includes most proportion of the second nucleotide binding domain of CFTR. The abnormal splicing may result in the absence of the important domain, which severely impairs the function of chloride channel.

Previous mutation screening of *CFTR* in CBAVD patients has mainly been performed in sporadic cases, and numerous heterozygous variants have been described. As the data have accumulated, some variants were proven to be quite common in patients as well as in the general population. The p.Phe508del variant was found in about 70% of northern European CBAVD patients (Tsui, 1990; Wagner, Zach, & Rosenkranz, 1992), while the IVS8‐5T variant was common in different populations with frequencies ranging from 20%–30% (Dayangac et al., 2004; Grangeia et al., 2005; Wu, Hsieh‐Li, Lin, & Chiang, 2004). Considering the conflict between the high number of *CFTR* variants and the low incidence of CBAVD, it is unlikely that one copy of the common or pathogenic variant alone is sufficient to cause CBAVD. Indeed, only one copy of the normal *CFTR* sequence can produce enough protein to maintain the normal function of the CFTR chloride channel. Instead, the combination of a common variant on one chromosome and a severe but rare variant on the other chromosome seems to be the major cause of CBAVD (Lissens et al., 1996).

In pedigree 1, both patients had bi‐allelic *CFTR* variants, one from their mother and the other likely from their father. Although their father has died, it can be inferred that he had a normal vas deferens because the children were conceived naturally. Therefore, one copy of the c.4056G>C;p.Gln1352Cys variant carried by the father could not cause CBAVD. In pedigree 2, the rarity of both variants suggests that they are more likely from each side of parents, although this cannot be validated in parents. However, when both male offspring inherited one variant from each parent, this resulted in CBAVD. Previous studies have reported bi‐allelic variants in some unrelated patients but this was not confirmed in their parents. Our study, in which compound heterozygous variants were identified in segregation with the disorder in a pedigree, provides the first known familial evidence that only recessive variants (homozygous or compound heterozygous) cause CBAVD.

Traditional sequencing of exons and splicing regions only detects one or no variants of *CFTR* in a large proportion of patients (Anzai et al., 2003; Li et al., 2012). The missing variants may reside in the regulatory regions of introns or be genetic rearrangements that escape detection by direct sequencing. The combination of next‐generation sequencing and bioinformatics may provide more comprehensive and accurate methods to thoroughly analyze the *CFTR* sequence, which could detect bi‐allelic pathogenic variants, especially those undetectable using traditional methods. The accurate detection of *CFTR* mutations is especially important for those patients who undergo assisted reproductive techniques, because the mutation may be transmitted to the offspring that causes increased risk of mild CF and CBAVD (de Souza, Faucz, Pereira‐Ferrari, Sotomaior, & Raskin, 2018). Therefore, comprehensive genetic counseling is strongly suggested for couples in which the male partner has CBAVD.

In conclusion, we identified compound heterozygous variants of *CFTR* in two Chinese pedigrees and provide more pedigree evidence that only recessive variants in *CFTR* can cause CBAVD. Our results suggest that whole exome sequencing may be an efficient method to identify variants in genes causing CBAVD.

## 5 ETHICS APPROVAL AND CONSENT TO PARTICIPATE

This research was approved by the Research Ethics Committee of Peking Union Medical College Hospital, and experiments were performed in accordance with approved guidelines.

## 6 CONSENT FOR PUBLICATION

Not applicable.

## 7 AVAILABILITY OF DATA AND MATERIAL

Not applicable.

## CONFLICT OF INTEREST

None of the authors declare competing financial interests.

## AUTHORS' CONTRIBUTION

YB analyzed and interpreted the data, drafted and revised the manuscript. WX and ZW collected the samples and performed the experiments. WBB and LHJ designed the study, revised and approved the manuscript.
